# Improved exercise ventilatory efficiency with nasal compared to oral breathing in cardiac patients

**DOI:** 10.3389/fphys.2024.1380562

**Published:** 2024-08-06

**Authors:** Prisca Eser, Pietro Calamai, Anja Kalberer, Laura Stuetz, Sarina Huber, Dominic Kaesermann, Sabina Guler, Matthias Wilhelm

**Affiliations:** ^1^ Centre for Rehabilitation and Sports Medicine, Inselspital, Bern University Hospital, University of Bern, Bern, Switzerland; ^2^ Department for Pulmonary Medicine, Allergology and Clinical Immunology, Inselspital, Bern University Hospital, University of Bern, Bern, Switzerland

**Keywords:** V_E_/VCO_2_ ratio, rapid shallow breathing index, exercise oscillatory ventilation, heart failure, nasal breathing

## Abstract

**Objectives:** To assess whether nasal breathing improves exercise ventilatory efficiency in patients with heart failure (HF) or chronic coronary syndromes (CCS).

**Background:** Exercise inefficient ventilation predicts disease progression and mortality in patients with cardiovascular diseases. In healthy people, improved ventilatory efficiency with nasal compared to oral breathing was found.

**Methods:** Four study groups were recruited: Patients with HF, patients with CCS, old (age≥45 years) and young (age 20–40 years) healthy control subjects. After a 3-min warm-up, measurements of 5 min with once nasal and once oral breathing were performed in randomized order at 50% peak power on cycle ergometer. Ventilation and gas exchange parameters measured with spiroergometry were analysed by Wilcoxon paired-sample tests and linear mixed models adjusted for sex, height, weight and test order.

**Results:** Groups comprised 15 HF, CCS, and young control and 12 old control. Ventilation/carbon dioxide production ( $\dot{V}$ _E_/ $\dot{V}$ CO_2_), ventilation ( $\dot{V}$ _E_), breathing frequency (f_R_), and end-tidal oxygen partial pressure (P_ET_O_2_) were significantly lower and tidal volume and end-tidal carbon dioxide partial pressure (P_ET_CO_2_) significantly higher during nasal compared to oral breathing in all groups, with large effect sizes for most parameters. For patients with HF, median $\dot{V}$ _E_/ $\dot{V}$ CO_2_ was 35% lower, f_R_ 26% lower, and P_ET_CO_2_ 10% higher with nasal compared to oral breathing, respectively. Exercise oscillatory ventilation (EOV) was present in 6 patients and markedly reduced with nasal breathing.

**Conclusion:** Nasal breathing during submaximal exercise significantly improved ventilatory efficiency and abnormal breathing patterns (rapid shallow breathing and EOV) in 80% of our patients with HF and CCS.

## Graphical Abstract

**Figure:**
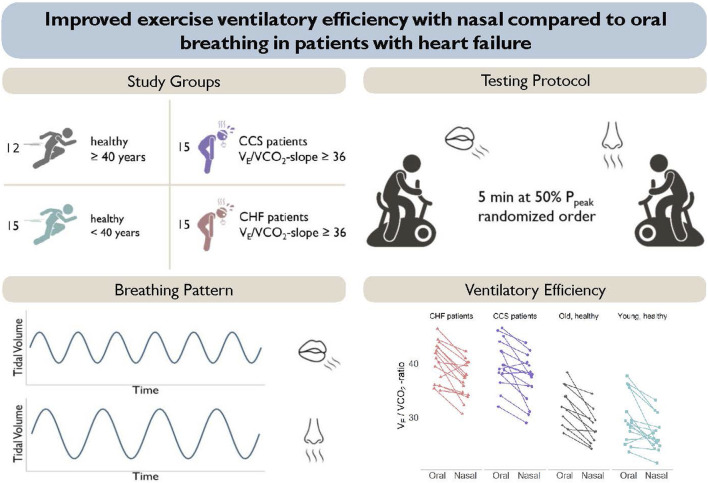
Presentation of the study groups and study protocol (top panels). Nasal breathing resulted in a pattern of slower and deeper breathing and a reduced ventilation to carbon dioxide production in all groups (lower panels).

## 1 Introduction

An exaggerated ventilatory response to exercise, often accompanied by early exertional dyspnea, is a hallmark in patients with chronic heart failure (HF) (Chua et al., 1996; Tomita et al., 2003). It has also been reported in patients with chronic coronary syndromes (CCS) and left ventricular dysfunction (Eser et al., 2023). Ventilatory inefficiency has not only been associated with reduced exercise capacity and quality of life but also with poorer prognosis (Ponikowski et al., 2001; Arena et al., 2004; Nadruz et al., 2017). It is quantified by an increased $\dot{V}$ _E_/ $\dot{V}$ CO_2_-slope, arising from an excessive rise of minute ventilation ( $\dot{V}$ _E_) with respect to carbon dioxide production ( $\dot{V}$ CO_2_) in the absence of metabolic acidosis (Agostoni and Guazzi, 2017). Based on the modified alveolar Eq. 1 an increased $\dot{V}$ _E_/ $\dot{V}$ CO_2_-slope can be explained by two factors: A reduced arterial CO_2_ partial pressure (P_a_CO_2_) and/or a high fraction of the tidal volume (V_T_) that goes to dead space (V_D_) (i.e., the V_D_/V_T_-ratio) (Wang et al., 2020).

$$\frac{V_{E}}{VCO_{2}}=\frac{863}{{P_{a}CO}_{2}*\left(1-\;\frac{V_{D}}{V_{T}}\right)}$$ (1)

Physiological dead space refers to the ventilated air that does not participate in gas exchange and is comprised of the anatomical dead space (i.e., the conducting airways) and the alveolar dead space (i.e., lung regions which are poorly perfused).

In patients with HF, impaired cardiac function may result in lung areas which are ventilated but poorly perfused (i.e., ventilation-perfusion mismatch) with $\dot{V}$ _E_ rising during exercise without sufficient rise in pulmonary perfusion (Weatherald et al., 2018). Furthermore, the V_D_/V_T_-ratio can be increased due to a reduced V_T_ during exercise when the diaphragm fatigues. Muscle fatigue in the diaphragm and/or the peripheral muscles leads to accumulating metabolites that trigger ergoreflexes which ellicit a steep increase in breathing frequency, (Piepoli et al., 1996), resulting in a pattern of rapid shallow breathing (RSB) (Weatherald et al., 2018). Increased chemosensitivity may further accelerate the abnormal ventilatory response to exercise in patients with HF (Chua et al., 1996; Ponikowski et al., 1997) and also in patients after acute myocardial infarction (Tomita et al., 2003). In patients with HF, the heart is enlarged and so restricts an appropriate increase in V_T_ (Cross et al., 2020). Consequently, in these patients ventilation is increased preferentially via an increase in breathing rate, again favoring a pattern of RSB.

Pharmaceutical as well as exercise therapies have been shown to reduce the exaggerated ventilatory response to exercise in patients with HF (Hambrecht et al., 1995). However, adherence to exercise recommendations may be poor in patients suffering from dyspnea, as exercise tolerance may be low (Cooper et al., 2015). There is an unmet need for further therapies to improve ventilatory efficiency and exercise tolerance. Slow breathing training has been shown to have positive effects on cardiorespiratory function, (Bernardi et al., 2002; Parati et al., 2008; Lachowska et al., 2019), and ventilatory efficiency (Parati et al., 2008) in patients with HF. Furthermore, in healthy volunteers it has been shown that nasal breathing can reduce the $\dot{V}$ _E_/ $\dot{V}$ CO_2_ ratio during exercise compared to oral breathing (Dallam et al., 2018; LaComb et al., 2017). Increased airway resistance leads to reduced breathing frequency, which allows more time for diffusion in the lungs and therefore better oxygenation (Dallam and Kies, 2020; Rappelt et al., 2023). This is supported by increased P_ET_CO_2_ and decreased end-tidal oxygen partial pressure (P_ET_O_2_) levels during nasal breathing (Dallam et al., 2018; Rappelt et al., 2023).

To date, no study has investigated whether nasal breathing is feasible in patients with HF or CCS and whether it is accompanied by a lower $\dot{V}$ _E_/ $\dot{V}$ CO_2E_/ $\dot{V}$ CO_2_-ratio compared to oral breathing. We aimed to close this gap in knowledge as these patients would be particularly prone to benefit from an improved breathing pattern and ventilatory efficiency during exercise.

The aims of the current study were to 1) Compare ventilatory efficiency and parameters of breathing pattern between oral and nasal breathing during submaximal exercise in patients with HF or CCS and inefficient ventilation; and 2) assess whether there is an age-related difference between oral and nasal breathing with regard to ventilatory efficiency by comparing healthy old volunteers (age-matched to the HF and CCS patients), and young healthy volunteers.

## 2 Methods

### 2.1 Study participants

This study was conducted as a sub-study of the Breathe-HF trial (NCT05057884). The sub-study included four different groups of participants, two cardiac patient groups and two healthy control groups. The inclusion and exclusion criteria are listed in Table 1. The rational for only including patients with $\dot{V}$ _E_/ $\dot{V}$ CO_2_ slope ≥36 was that this parameter is an objective criteria reflecting exercise induced dyspnea. Our study intervention of slow nasal breathing training aimed to improve exercise breathing efficiency.

**TABLE 1:** In- and exclusion criteria of the different groups’ study participants.

Group	In-/Exclusion criteria
CHF patients	Inclusion criteria
	• Diagnosis based on current guidelines, including CHF with either preserved or reduced ejection fraction^23^
	• V̇_E_/V̇CO_2_-slope ≥36
	• New York Heart Association functional classes II or III
	• 18-80 years
	• Optimal guideline-directed therapy and stable disease state during previous 3 months
	Exclusion criteria
	• Non-cardiac conditions associated with hyperventilation
	• Heart transplant
	• Pregnant or lactating women
CCS patients	Inclusion criteria
	• Diagnosis based on current guidelines^23^
	• V̇_E_/V̇CO_2_-slope ≥36
	• 18-80 years
	• Optimal guideline-directed therapy and stable disease state (no acute coronary syndrome) during the preceding 4 weeks
	Exclusion criteria
	• Non-cardiac conditions associated with hyperventilation
	• Heart transplant
	• Pregnant or lactating women
Old healthy control subjects	Inclusion criteria
	• Aged 39-80, healthy
	Exclusion criteria
	• present or past smoking
	• present or past cardiovascular or pulmonary disease
	• present consumption of blood pressure or asthma medication
Young healthy control subjects	Inclusion criteria
	• Aged 18-39, apparently healthy
	Exclusion criteria
	• present or past smoking
	• present or past cardiovascular disease
	• present consumption of blood pressure or asthma medication

CHF, chronic heart failure; CCS, chronic coronary syndrome; V̇_E_/V̇CO_2_, ventilation to carbon dioxide production slope.

### 2.2 Study procedures

Eligible patients with HF and CCS were identified and recruited during their yearly check-up visit at a tertiary university referral centre. Healthy young and old volunteers were recruited by word of mouth. If they met the inclusion criteria and consented in writing, they were included in the study and measurements were performed as summarized in Supplementary Figure S1. The study was approved by the ethics committee of the Canton of Berne.

Body composition was assessed by bioelectrical impedance with a body composition analyzer (inbody 770, best4health gmbh, Bassersdorf, Switzerland). Weight, lean muscle mass, and body fat percentage were measured and reported for comparison of anthropometric characteristics between groups. Moreover, body mass index (BMI) was calculated.

### 2.3 Cardiopulmonary exercise testing

Exercise capacity was assessed with a CPET on a cycle ergometer. Prior to the test, a vital capacity (FCV, l) and forced expiratory volume in one second (FEV_1_, l*min^-1^) was assessed by spirometry. Then, after sitting on the ergometer quietly for 3 min, blood pressure was measured two times and the lowest measurement was recorded. A 3 min warm-up was followed by an individually set ramp as previously described (Eser et al., 2022). Volumes, flows and gases were sampled continuously in an open spirometric system (Quark, Cosmed, Rome, Italy) and averaged over 8 breaths, as recommended (Glaab and Taube, 2022). Measured variables included oxygen uptake ( $\dot{V}$ O_2_, ml*min^−1^*kg^−1^), carbon dioxide production ( $\dot{V}$ CO_2_ ml*min^−1^), minute ventilation ( $\dot{V}$ _E_, l*min^−1^), respiration frequency (f_R_, breaths*min^−1^), tidal volume (V_T_, l) and end-tidal partial pressures of O_2_ (P_ET_O_2_, mmHg) and CO_2_ (P_ET_CO_2_, mmHg), heart rate (HR, beats*min^-1^) and oxygen saturation (SpO_2_, %). Additionally, the respiratory exchange ratio (RER = $\dot{V}$ CO_2_/ $\dot{V}$ O_2_) and the oxygen pulse ( $\dot{V}$ O_2_/heart beat) were calculated. $\dot{V}$ O_2peak_ (ml*min^−1^*kg^−1^) was defined as the highest value of oxygen consumption averaged over 30 s. The first (VT1) and second ventilatory threshold (VT2) were identified using the Wassermann’s 9-panel plot (Marcin et al., 2020). The $\dot{V}$ _E_/ $\dot{V}$ CO_2_-slope was determined from the start of the ramp until VT2. Further, the nadir of the $\dot{V}$ _E_/ $\dot{V}$ CO_2_-ratio was defined as the lowest $\dot{V}$ _E_/ $\dot{V}$ CO_2_-ratio during exercise.

### 2.4 Oral and nasal submaximal tests

After the CPET following a 15-min resting period (or on a separate day if the CPET was performed as part of clinical routine testing), all subjects completed a submaximal constant load cycling protocol with exclusively oral and nasal breathing in randomized order. The protocol consisted of a 3 min warm-up phase followed by 5 min of constant load cycling at 50% of peak power output. This intensity was chosen based on a study by LaComb (LaComb et al., 2017) and the fact that some people have difficulties breathing through their nose at high intensities. During the oral breathing subjects were required to wear a nose clip under the mask, whereas during the nasal breathing their mouth was covered with tape.

Participants were instructed to always maintain a cadence of 60–70 min^−1^. The two trials were separated by a 10-min break to allow for some recovery. Rating of perceived exertion (RPE) was noted upon completion of each trial in the patient groups. All parameters of breathing patterns and gas exchange were calculated as averages of the fifth minute. The rapid shallow breathing index (RSBI, m^2^*min^−1^* l^−1^) was calculated by dividing f_R_ by V_T_. Exercise ventilatory oscillation (EOV) was defined according to guidelines (Guazzi et al., 2012).

### 2.5 Statistical analysis

All analyses were performed by R (R Core Team, 2021; Version 4.1.0). Primary outcome was the within-subject difference in $\dot{V}$ _E_/ $\dot{V}$ CO_2_-ratio between oral and nasal breathing modes. This was analysed by within group differences between oral and nasal breathing using paired Wilcoxon tests. Further, the effect of breathing mode was analysed by the group × mode interaction effect of a linear mixed model with package “nlme” (Version 3.1–152) with fixed effects breathing mode, patient/volunteer group, order of the nasal breathing mode, adjusted for sex, height and weight. Adjustment for age was not included as the effect of age was investigated by including an age-matched and a young control group. Subjects were entered as random intercepts. Effect sizes were calculated by the package “sjPlot” (version 2.8.6). Secondary outcomes were the differences between oral and nasal breathing in $\dot{V}$ e, V_T_, f_R_, P_ET_O_2_, P_ET_CO_2_, RSBI, $\dot{V}$ O_2_, HR, and $\dot{V}$ O_2_/HR averaged over the 5^th^ minute, all analyzed using linear mixed models as specified for the primary outcome. Old control subjects, oral breathing, first order and female patients were set as reference categories.

Baseline characteristics were tested between groups by Kruskal-Wallis tests followed by *post hoc* testing (only patient groups and young control subjects were tested against old control subjects) adjusted for multiple testing by Benjamini-Hochberg correction. Categorical variables were tested by Fisher’s exact tests. Statistical significance for all tests was set at a *p*-value <0.05.

## 3 Results

### 3.1 Study population

**FIGURE 1:**
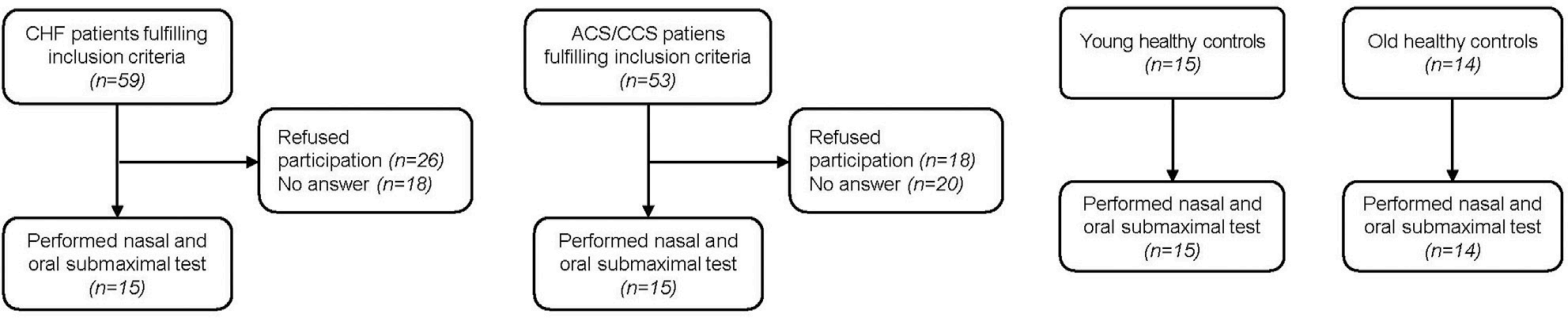
Patient/participant flow of the four groups.

Fifteen young and 14 old healthy control subjects were recruited for the present study (Figure 1). Of patients with HF performing CPETs for yearly clinical visits, 59 qualified for inclusion. Eighteen could not be reached by phone and 26 declined participation in the study. Within the HF group, eleven patients were classified as having reduced (HFrEF), three as having mildly reduced (HFmrEF) and one as having preserved ejection fraction (HFpEF) (McDonagh et al., 2021). Of 53 patients with CCS qualifying for the study, 20 could not be contacted and 18 declined participation, leaving 15 who participated in the study. Two old healthy controls had a blocked nose and had to stop the nasal trial after 3 min, leaving data from 12 old healthy controls in the analyses. Otherwise the exercise bouts with the different breathing modes were tolerated well. There were no significant differences between old healthy control subjects and the two patient groups with regard to baseline characteristics (Table 2). The only significantly different baseline characteristics were age and percent body fat between old and young healthy control subjects.

**TABLE 2:** Baseline characteristics of included subjects. Shown are medians and first and third quartiles in round brackets for each group.

	CHF patients (n = 15)*	CCS patients (n = 15)*	Old control subjects (n = 12)	Young control subjects (n = 15)
Age	68 (63, 71)	68 (63, 73)	63 (58, 72)	25 (24, 30)^a^
Male/Female	11/4	12/3	8/4	9/6
Weight [kg]	79.9 (67.4, 84.7)	78.8 (72.0, 83.6)	72.5 (63.9, 84.0)	68.2 (63.4, 73.9)
Height [cm]	173 (164, 182)	175 (167, 179)	178 (165, 182)	173 (169, 180)
BMI [kg/m^2^]	26.2 (23.4, 28.8)	27.9 (23.1, 29.2)	24.8 (22.2, 25.7)	22.2 (21.0, 23.6)
Lean muscle mass [kg]*	34.4 (31.8, 40.2)	32.8 (30.2, 34.0)	30.3 (26.5, 36.7)	32.5 (28.6, 35.8)
Percent body fat [%]*	26.4 (16.2, 30.6)	30.1 (23.5, 33.4)	22.4 (19.8, 24.8)	16.9 (13.2, 18.8)^a^
Systolic blood pressure	110 (100, 118)	120 (115, 132)	120 (113, 120)	117 (110, 120)
Diastolic blood pressure	70 (63, 70)	75 (65, 80)	80 (78, 83)	80 (76, 80)
LV ejection fraction [%]	40.0 (35.8, 47.0)	59.8 (56.7, 63.5)	-	-
History of acute coronary syndrome	7	10	-	-
History of percutaneous coronary intervention	9	11	-	-
Cardiac implantable electric device	9	1	-	-

The following indices mark adjusted p-value <0.05 of post hoc Kruskal Wallis tests between old control subjects and other groups: ^a^ between old and young control subjects.

CHF, chronic heart failure; CCS, acute/chronic coronary syndrome; BMI, body mass index; LV, left ventricular

*Data missing from one CCS and six CHF patients due to inability to conduct body composition measurement because of Cardiac implantable electric device (CIED).

### 3.2 Results of cardiopulmonary exercise tests

Resting parameters of the two patient groups were comparable to old control subjects except for P_ET_CO_2_ that was lower in patient with CCS (Supplementary Table S1). The first ventilatory threshold occurred at lower power, lower VO_2_, lower $\dot{V}$ _E_ and V_T_ relative to body weight in both patient groups compared to old control subjects. Based on the inclusion criteria for both patient groups, they had significantly higher $\dot{V}$ _E_/ $\dot{V}$ CO_2_-slopes and nadir $\dot{V}$ _E_/ $\dot{V}$ CO_2_. At peak exercise, power, VO_2,_ P_ET_CO_2_ and HR were lower than in the old control subjects (Supplementary Table S1).

### 3.3 Nasal compared to oral breathing

Non-parametric data of ventilatory and circulatory parameters during the submaximal cycling with nasal and oral breathing modes of the four different groups are shown in Table 3. Power output was constant between nasal and oral breathing and was comparable to power at VT1 (range −14% to 8%), indicating that the tests were completed during aerobic metabolism. Subjectively perceived exertion did not differ between breathing modes.

**TABLE 3:** Ventilatory and circulatory parameters during 5 minutes of submaximal cycling with exclusively nasal or oral breathing. The intensity was set at 50% of their peak power achieved during the CPET. For each participant values were averaged over the 5^th^ minute. Shown are medians and first and third quartiles in round brackets for each group and each condition in randomized order.

	CHF patients (n = 15)		CCS patients (n = 15)		Old control subjects (n = 12)		Young control subjects (n = 15)	
Power at 50% [watt]	55.0 (42.3, 64.0)		70.0 (57.0, 90.0)		113 (103, 132)		151 (121.3, 170)	
Power at 50% [watt*kg^-1^]	0.71 (0.65, 0.83)		0.88 (0.77, 0.96)		1.73 (1.35, 2.10)		2.24 (1.95, 2.51)	
Difference to power at VT1	-14% (-19%, 17%)		4% (-11%, 18%)		-6% (-22%, 0%)		8% (-19%, 9%)	
	Oral	Nasal	Oral	Nasal	Oral	Nasal	Oral	Nasal
V̇_E_ [l⋅min^-1^]	45.3 (42.8, 49.2)	44.1 (37.7, 51.2)	50.7 (44.1, 58.0)	47.7 (42.2, 52.5)^†^	54.3 (51.9, 62.9)	46.7 (43.5, 54.3)^†^	59.8 (57.1, 67.1)	60.3 (52.1, 62.7)*
f_R_ [min^-1^]	30.0 (25.9, 31.3)	22.2 (20.7, 24.8)^†^	27.9 (25.2, 30.1)	23.8 (21.3, 25.8)^‡^	27.5 (22.1, 30.2)	22.1 (18.0, 23.6)^†^	29.9 (25.5, 32.9)	22.8 (20.4, 27.0)^†^
V_T_ [l]	1.61 (1.37, 1.88)	1.73 (1.55, 2.36)^‡^	1.96 (1.66, 2.14)	2.01 (1.70, 2.30)	2.17 (1.88, 2.43)	2.37 (2.07, 2.79)*	2.19 (1.96, 2.47)	2.50 (2.28, 2.94)^‡^
RSBI [breaths l^-1^ min^-1^]	19.4 (14.4, 24.1)	12.7 (9.0, 16.1)^†^	15.2 (12.0, 17.8)	11.6 (8.9, 14.9)^†^	12.3 (9.7, 15.3)	9.6 (6.4, 11.3)*	12.5 (10.6, 16.1)	8.5 (7.3, 12.9)^†^
P_ET_CO_2_ [mmHg]	29.1 (28.2, 31.3)	32.2 (30.4, 34.0)^‡^	29.5 (28.5, 31.3)	31.3 (30.6, 35.4)^‡^	36.4 (33.2, 38.2)	42.6 (38.7, 45.2)^‡^	37.7 (33.8, 40.3)	42.2 (39.0, 45.2)^†^
P_ET_O_2_ [mmHg]	108 (106, 111)	106 (102, 108)^†^	110 (104, 110)	104 (102, 108)^‡^	100 (97.1, 102)	92.5 (88.4, 97.2)^‡^	99.4 (96.3, 104)	94.8 (92.6, 98.4)^‡^
V̇_E_/V̇CO_2_-ratio	40.9 (36.7, 43.0)	37.4 (34.4, 39.9)^‡^	39.7 (38.2, 43.2)	37.8 (34.6, 39.9)^‡^	31.5 (28.5, 34.5)	27.4 (25.1, 29.1)^‡^	28.6 (26.7, 33.1)	25.8 (24.8, 27.8)^†^
V̇O_2_ [ml⋅kg^-1^⋅min^-1^]	14.5 (13.4, 15.5)	15.6 (14.2, 17.5)^†^	15.9 (14.9, 17.9)	16.7 (15.7, 18.6)	27.5 (23.1, 29.7)	28.5 (24.6, 31.6)	32.8 (31.2, 36.2)	33.9 (31.8, 39.1)
V̇CO_2_ [ml⋅kg^-1^⋅min^-1^]	13.07 (12.05, 15.08)	13.93 (12.60, 17.16)^‡^	15.01 (14.04, 17.11)	15.47 (14.51, 17.50)	23.07 (20.64, 27.21)	23.77 (20.94, 28.00)	31.72 (28.09, 33.72)	32.21 (29.12, 34.90)
RER	0.91 (0.88, 0.92)	0.91 (0.87, 0.96)	0.96 (0.91, 1.01)	0.93 (0.89, 0.98)	0.90 (0.87, 0.93)	0.88 (0.86, 0.91)	0.92 (0.86, 0.98)	0.91 (0.88, 0.96)
HR [bpm]	89.0 (80.5, 101)	88.6 (83.9, 106)*	99.0 (95.0, 108)	102 (94.2, 121)	130 (123, 145)	130 (124, 148)	149 (141, 162)	148 (144, 163)
V̇O_2_/HR [ml/heart beat]	12.6 (10.7, 16.1)	13.1 (11.2, 16.4)	12.0 (10.6, 14.6)	12.3 (10.9, 13.5)	14.6 (13.1, 15.3)	14.7 (12.7, 16.6)*	14.6 (12.5, 17.1)	15.7 (13.2, 17.2)
RPE^a^	13.0 (11.5, 13.0)	13.0 (12.0, 13.0)	12.0 (11.8, 13.0)	12.0 (11.5, 13.0)	NA	NA	NA	NA

The following indices mark p-values of within group Wilcoxon paired sample tests between nasal and oral breathing: *, p≤0.05; ^†^, p≤0.01, ^‡^, p≤0.001

^a^Data missing from two CHF patients

CHF, chronic heart failure; CCS, acute/chronic coronary syndrome; VT1, first ventilatory threshold; V̇e, minute ventilation; f_R,_ breathing frequency; V_T_, tidal volume*;* P_ET_CO_2,_ end-tidal partial pressure of CO_2_; P_ET_O_2_, end-tidal partial pressure of O_2_; V̇O_2_, oxygen consumption; V̇CO_2_, carbon dioxide production; RER, respiratory quotient (V̇CO_2_/V̇O_2_); HR, heart rate

**FIGURE 2:**
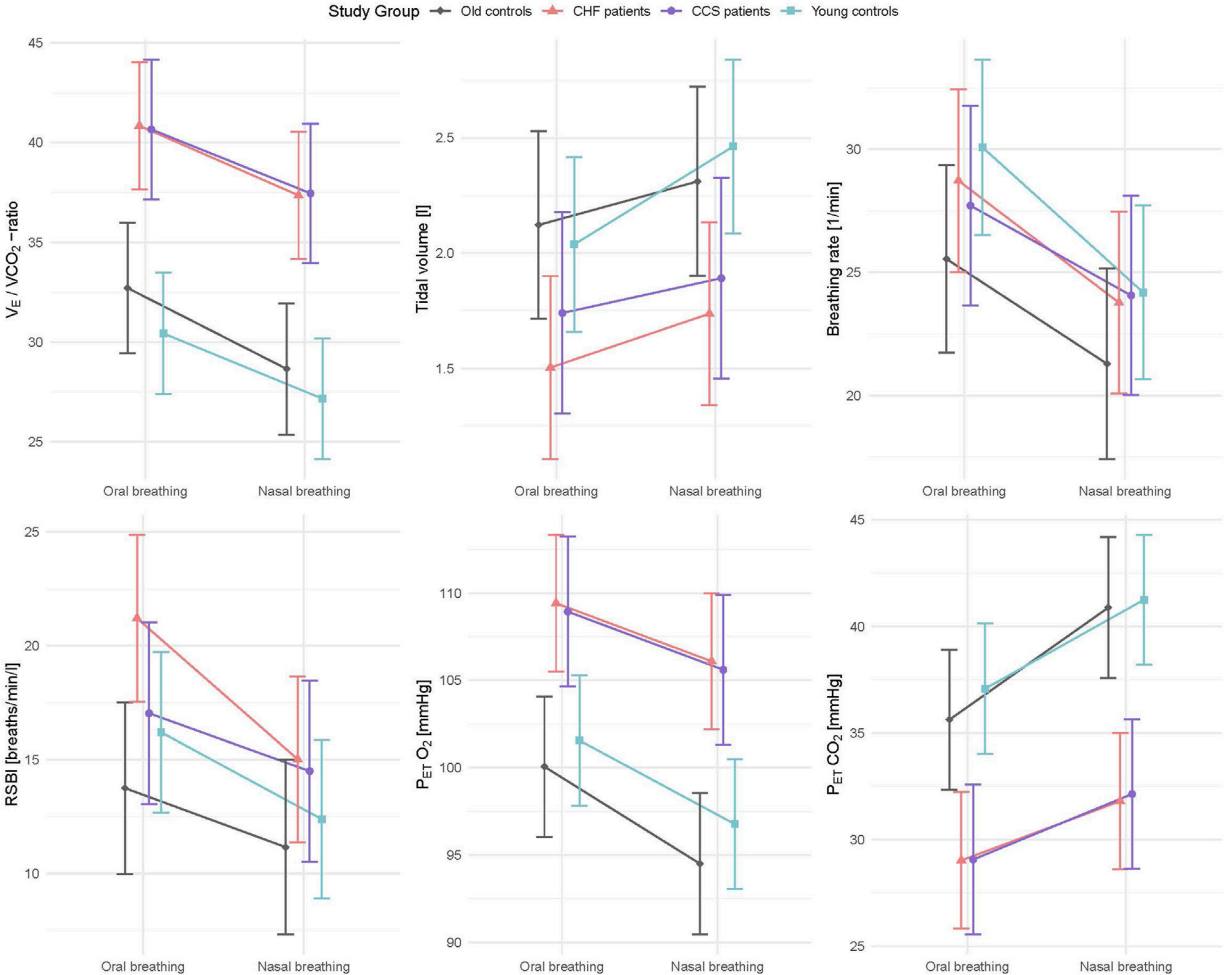
Interaction plots of effects of breathing modes and groups on $\dot{V}$ _E_/ $\dot{V}$ CO_2_ slope, V_T_, f_B_, RSBI, P_ET_O_2_ and P_ET_CO_2_ adjusted for order, sex, height and weight. CHF, chronic heart failure; CCS, acute/chronic coronary syndrome; $\dot{V}$ _E_/ $\dot{V}$ CO_2_, ventilation to carbon dioxide production; V_T_, tidal volume; f_B_, breathing frequency; RSBI, rapid shallow breathing index; P_ET_O_2_, end tidal partial pressure of oxygen: P_ET_CO_2_, end tidal partial pressure of CO_2_.

**FIGURE 3:**
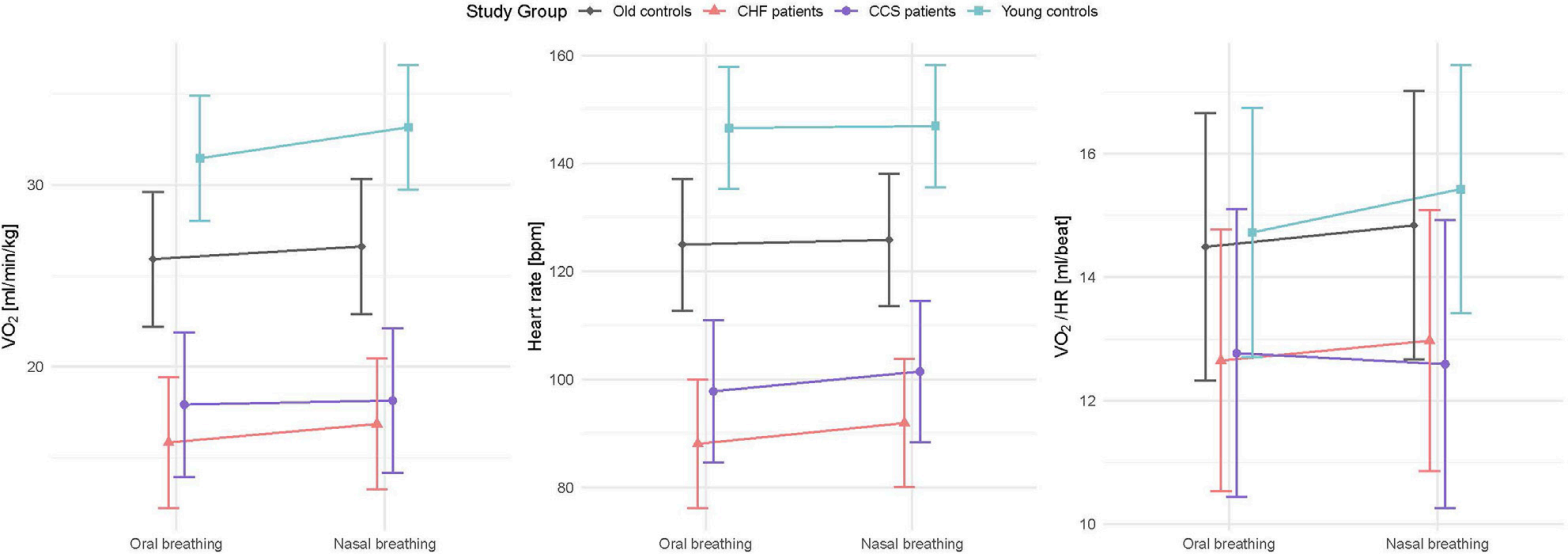
Interaction plots of effects of breathing modes and groups on $\dot{V}$ O_2_, HR and oxygen pulse ( $\dot{V}$ O_2_/HR) adjusted for order, sex, height and weight. HF, chronic heart failure; CCS, acute/chronic coronary syndrome; $\dot{V}$ O_2_, oxygen consumption; HR, heart rate; $\dot{V}$ O_2_/HR, oxygen pulse.

**FIGURE 4:**
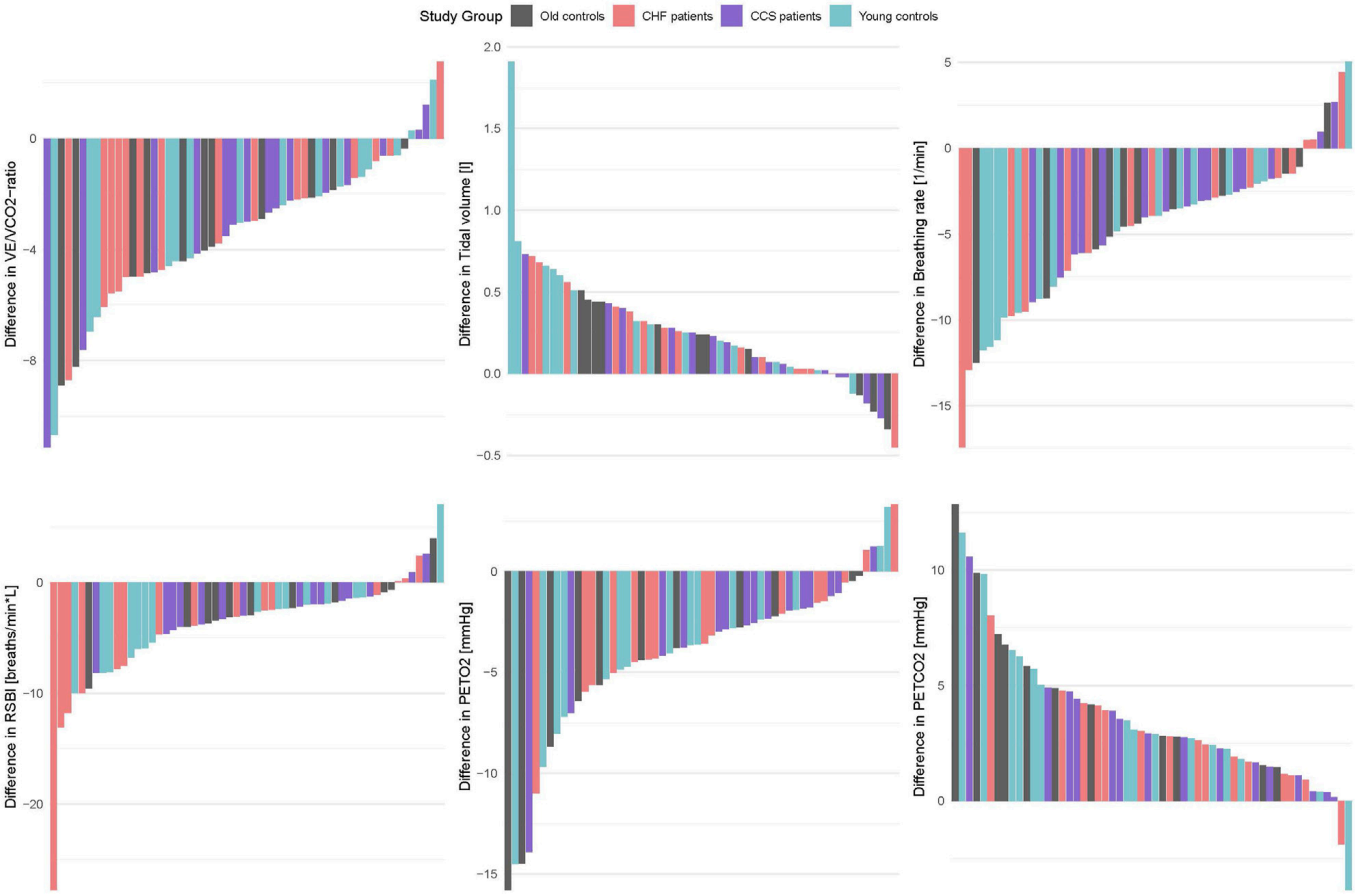
Barplots of within-subject differences between oral and nasal breathing (oral value–nasal value) for the same parameters as shown in Figure 2.

Mean values during oral and nasal breathing of all primary and secondary outcome variables ( $\dot{V}$ _E_/ $\dot{V}$ CO_2_-ratio, $\dot{V}$ _E_, V_T_, f_R_, $\dot{V}$ O_2_, P_ET_O_2_, P_ET_CO_2_, HR and $\dot{V}$ O_2_/HR) analysed by linear mixed models are displayed in Figures 2, 3. Nasal breathing led to strong effect sizes in lowering $\dot{V}$ _E_/ $\dot{V}$ CO_2_-ratio (standardized beta −0.64, *p* < 0.001), $\dot{V}$ e (−0.52, *p* = 0.004) and f_R_ (−0.78, *p* < 0.003) and small effect size for increasing V_T_ (0.29, *p* = 0.083, Supplementary Table S2). Specifically, median V_T_ was raised by nasal breathing significantly in all groups except old controls (Table 3). There was no effect on breathing mode on $\dot{V}$ O_2_ in the old control subjects (Supplementary Table S2 and Figure 3), however, in the HF group median $\dot{V}$ O_2_ was higher by 1.1 mL/kg/min or 7.6% (*p* = 0.007, Table 3). Lower f_R_ and higher V_T_ lead to a reduced RSBI (−0.41, *p* = 0.084, Supplementary Table S2 and Figure 2), whose median was reduced by 34.5% (*p* < 0.01) in the HF group (Table 3). Likewise, P_ET_CO_2_ levels were significantly higher (0.88, *p* < 0.001) and P_ET_O_2_ levels lower (−0.80, *p* < 0.001) in all groups (Supplementary Table S2 and Figure 2), albeit to a smaller degree in the patient groups. Within-subject differences between nasal and oral breathing modes are shown in Figure 4. HR was also not different between the two breathing modes and neither was oxygen pulse, the ratio between $\dot{V}$ O_2_ and HR (Table 3, Figure 3).

**FIGURE 5:**
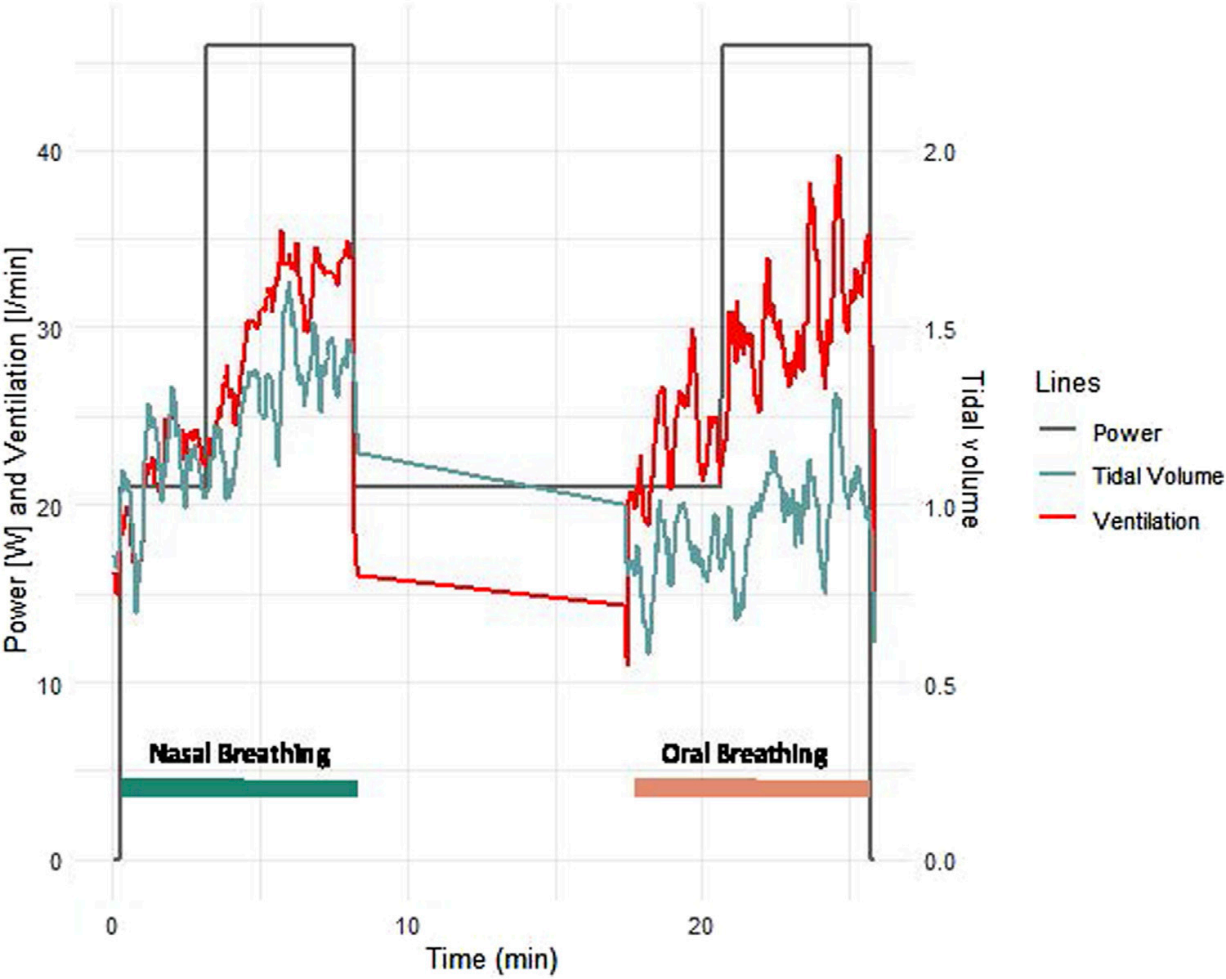
Example of ventilation and tidal volume of a typical female 63 years old patient with heart failure with reduced ejection fraction. The mask was not worn during the 10 min rest phase between exercise bouts.

Patients with CCS had similar values to patients with HF for all measured parameters and similar improvements with nasal breathing (Figures 2, 3). Five patients with HF and one patient with CCS had EOV during oral breathing which in all these patients was markedly dampened with nasal breathing (Figure 5).

Based on selection criteria, the young and old healthy group had lower $\dot{V}$ _E_/ $\dot{V}$ CO_2_-ratio during both breathing modes (Graphical abstract). Associated with this higher ventilatory efficiency were lower P_ET_O_2_ levels and higher P_ET_CO_2_ levels. Patients with HF had lower V_T_ (*p* = 0.003) and patients with CCS tended to have lower V_T_ (*p* = 0.062) than old healthy volunteers (Supplementary Table S2). Sex was not a significant factor in any of the models (Supplementary Table S2).

## 4 Discussion

Our study is the first to demonstrate that nasal breathing could reduce the excessive ventilatory response to exercise (represented by a lower $\dot{V}$ _E_/ $\dot{V}$ CO_2_-ratio) by significantly lowering f_R_ and increasing V_T_, leading to a greatly reduced RSBI in patients with HF and CCS. It is noteworthy that 93% of patients with CHF and 80% of those with CCS improved their ventilatory efficiency with nasal compared to oral breathing (and 93% in controls, for summary see Figure 6). Concomittantly, P_ET_CO_2_ levels were raised and P_ET_O_2_ levels reduced, indicating improved gas exchange likely due to reduced physiologic dead space. In 6 patients with EOV during oral breathing, nasal breathing markedly reduced the amplitudes in ventilation. The differences between breathing modes had large effect sizes and were consistent in all groups, suggesting that the difference between nasal and oral breathing is not disease-specific and independent of age.

Nasal breathing compared to oral breathing lowered $\dot{V}$ _E_ and f_R_ and increased V_T_ in all groups. As a consequence, RSBI was reduced by 34.5% with nasal breathing in the HF group, indicating that abnormal breathing patterns can be largely normalized in these patients (reaching almost the median of the old control subjects with oral breathing, Table 2). RSB increases anatomical dead space, with the consequence that P_ET_CO_2_ increasingly underestimates P_a_CO_2_ (McSwain et al., 2010). Dead space is composed of anatomical and physiologic dead space, with the latter being large due to ventilation-perfusion mismatch based on reduced perfusion and/or edematous lung parenchyma in heart patients (Cross et al., 2020). Anatomical dead space of nasal breathing has been found to be approximately 0.03 L greater than oral breathing (Tanaka et al., 1985). An increased anatomical dead space has been found to lead to increased ventilation (Ward and Whipp, 1980). Contrarily and in accordance with Douglas and colleagues (Douglas et al., 1983) we found a decrease in ventilatory drive with nasal as compared to oral breathing. While we cannot calculate dead space in the absence of blood gas analyses, the improvement of ventilatory efficiency in all patients with CHF and over 90% of all subjects tested in the present study indicates a reduction rather than an increase in pulmonary dead space.

In our CHF group, median $\dot{V}$ _E_/ $\dot{V}$ CO_2_ was reduced by 3.6, which is comparable to the reduction of 3.7 found with a 3-month thrice weekly high-intensity interval training (HIIT), (Donelli da Silveira et al., 2020), greater than the 2.0 found in a similar study (Iellamo et al., 2013) but less than the 5.3 found in a 16-week thrice weekly HIIT (Smart and Steele, 2012). Ventilatory efficiency was improved by 9% in our patients with HF by an acute bout of nasal breathing, which compares well against the achieved 14% by 6 months of taking enalapril (Guazzi et al., 1999).

Our results are consistent with findings of previous studies in healthy individuals, where acute nasal breathing was found to reduce $\dot{V}$ _E_/ $\dot{V}$ CO_2_-ratio by approximately 10% compared to acute oral breathing (Dallam et al., 2018; LaComb et al., 2017).

$\dot{V}$ _E_ and f_R_ were reduced with nasal breathing most likely as a result of increased airway resistance, as has been previously shown in healthy volunteers (Rappelt et al., 2023; Shi et al., 1999). Since median $\dot{V}$ O_2_ were higher with nasal breathing in all groups, this most likely lead to also higher $\dot{V}$ CO_2_, which would have partly been responsible for the reduced $\dot{V}$ _E_/ $\dot{V}$ CO_2_ ratio. The greater V_T_ probably caused a greater oxygen demand by the diaphragm and other breathing muscles. In contrast, in the studies by Dallam and colleagues as well as by Rappelt and colleagues $\dot{V}$ O_2_ was decreased with nasal breathing during steady state submaximal exercise compared to oral breathing, suggesting that less metabolic energy was required to complete the same work (Dallam et al., 2018; Rappelt et al., 2023). We suggest that differences in oxygen demand between their and our study may be explained by the fact that their subjects were selected from a population of recreational athletes well adapted to nasal breathing (performing their regular running training with nasal breathing) while our subjects were not specifically selected with regard to habitual breathing mode. It is possible that adequate training of the diaphragm may be needed to adapt the diaphragm to the changed force-length relationship at a greater V_T_ in order to achieve adequate economization of energy demand and hence $\dot{V}$ O_2_. On the other hand, lower lung compliance in patients with HF (Cross et al., 2020) would also explain the higher oxygen demand by the diaphragm with increasing V_T_. Despite the higher $\dot{V}$ O_2_ with nasal breathing in our subjects, respiratory quotient and RPE did not differ between nasal and oral breathing.

Potential mechanisms underlying the improvement of ventilatory efficiency with nasal breathing may be the alteration of breathing pattern with lower f_R_ and increased V_T_, with the latter having been shown to reduce muscle sympathetic neural activation in some (Hering et al., 2013; Oneda et al., 2010) but not all studies (Limberg et al., 2013). Another potential mechanism reducing $\dot{V}$ _E_/ $\dot{V}$ CO_2_ with nasal breathing may be the airflow (particularly with cool air) through the nose that stimulates upper airway receptors which have been shown to dampen ventilatory drive (McBride and Whitelaw, 1981).

The increase in P_ET_CO_2_ and decrease in P_ET_O_2_ with nasal breathing indicates either a more efficient oxygen extraction or a better reflection of alveolar partial pressures of CO_2_ and O_2_, based on the fact that breathing at higher f_R_ increases the ratio of air that goes to anatomical dead space. Further, nasal breathing has been suggested to lead to higher nitric oxide (NO) concentrations in the inhaled air than oral breathing as the main production site of NO, the paranasal sinuses, are circumvented by oral breathing (Lundberg et al., 1996; Lundberg et al., 1995). Nasal breathing has been shown to reduce pulmonary vascular resistance compared to oral breathing in an invasive study in patients with HF (Settergren et al., 1998). Inhaled NO, which is recommended intraoperatively in patients with pulmonary hypertension for selective pulmonary vasodilation, (Rajagopal et al., 2023), and during cardiopulmonary bypass, (Abouzid et al., 2023), has been shown to improve ventilation-perfusion matching (Dembinski et al., 2000; Hoffman and Nelin, 2005; Hajian et al., 2016).

It has been suggested that the rapid shallow breathing pattern may be adapted by patients with HF to avoid large intrathoracic pressure swings to preserve cardiac output (Lalande and Johnson, 2010). In our study, the O_2_ pulse, an accepted surrogate parameter for stroke volume, was not found to be different between nasal and oral breathing in neither of our patient groups, so we cannot confirm that a greater V_T_ leads to a reduction of stroke volume in these patients.

Strengths of the present study were the inclusion of a representative cohort of well phenotyped patients with CHF and CCS and a rigorous within-subject study protocol, CPET based measurements, and random assignment of the order of the intervention to each study participant. Further, the inclusion of both sexes as well as a young healthy control group showed that the found effects of nasal breathing were independent of sex and age and similarly applied to all groups.

In the present study, we set the intensity at 50% of the maximal power despite results by LaComb and colleagues in healthy people suggesting that the effects of nasal breathing on reducing $\dot{V}$ _E_/ $\dot{V}$ CO_2_-ratio may be greater at intensities higher than 50% (LaComb et al., 2017). However, 50% of maximal power output corresponded to the first ventilatory threshold and hence for the intensity recommended for patients with HF and CCS (McDonagh et al., 2021; Knuuti et al., 2020).

The main limitation of our study was the lack of blood gas analyses due to logistic reasons, which prevented calculation of dead space. A further limitation was that no dyspnea perception rating was included, so we can only assume from the RPE that patients subjectively felt the same amount of dyspnea during both trials. Also, the chosen bout duration was 5 min in this study for logistic reasons so that all patients managed to perform two bouts during one visit to the lab. Whether the same differences between breathing modes would result from longer bouts would have to be tested in a more involving study protocol with bouts on separate days. Last but not least, since we only included patients with $\dot{V}$ _E_/ $\dot{V}$ CO_2_ slopes ≥36, we cannot extrapolate our findings to patients with only mildly increased ventilatory inefficiency, however, the consistency of our findings across all our groups suggests that the effect of nasal breathing would be similar.

We conclude that in healthy subjects and patients with HF or CCS alike, nasal breathing led to a reduced $\dot{V}$ _E_/ $\dot{V}$ CO_2_ ratio, reduced f_R_ and increased V_T_ compared to oral breathing at moderate exercise intensity. The improved gas exchange and breathing pattern in patients with HF or CCS suggests that nasal breathing should be the recommended breathing mode at moderate exercise intensities in patients with inefficient ventilation.

## Data Availability

The raw data supporting the conclusions of this article will be made available by the authors, without undue reservation.

## Supplementary material

SUPPLEMENTARY FIGURE S1 Protocol of study measurements comparing oral and nasal breathing at 50% or peak power output that was determined during a preceding maximal ramp test. *order of oral and nasal breathing was set randomly.

## Abbreviations

BMI, body mass index; BSA, body surface area; CCS, chronic coronary syndrome; CO_2_, carbon dioxide; CPET, cardiopulmonary exercise test; EOV, exercise oscillatory ventilation; FCV, forced vital capacity; FEV_1_, forced expiratory volume in the first second; f_R_, respiratory frequency; HF, heart failure; HFpEF; heart failure with preserved ejection fraction; HFrEF, heart failure with reduced ejection fraction; HR, heart rate; O_2_, oxygen; P_a_CO_2_, arterial partial pressure of carbon dioxide; P_a_O_2_, arterial partial pressure of oxygen; P_ET_CO_2,_ end-tidal partial pressure of carbon dioxide; P_ET_O_2_, end-tidal partial pressure of oxygen; RER, $\dot{V}$ CO_2_/ $\dot{V}$ O_2_; RPE, rate of perceived exertion; RSBI, rapid shallow breathing index; SpO_2_, oxygen saturation; V_D_, pulmonary dead space; $\dot{V}$ _E_, ventilation; $\dot{V}$ CO_2_, carbon dioxide production; $\dot{V}$ O_2_, oxygen production; V_T_, tidal volume; VT1, first ventilatory threshold, VT2, second ventilatory threshold.
