# Corrigendum: Improved exercise ventilatory efficiency with nasal compared to oral breathing in cardiac patients

**DOI:** 10.3389/fphys.2024.1516510

**Published:** 2024-11-05

**Authors:** Prisca Eser, Pietro Calamai, Anja Kalberer, Laura Stuetz, Sarina Huber, Dominic Kaesermann, Sabina Guler, Matthias Wilhelm

**Affiliations:** ^1^ Centre for Rehabilitation and Sports Medicine, Inselspital, Bern University Hospital, University of Bern, Bern, Switzerland; ^2^ Department for Pulmonary Medicine, Allergology and Clinical Immunology, Inselspital, Bern University Hospital, University of Bern, Bern, Switzerland

**Keywords:** VE/VCO_2_ ratio, rapid shallow breathing index, exercise oscillatory ventilation, heart failure, nasal breathing

In the published article, **Authors**’ names were incorrectly written as “Eser Prisca, Calamai Pietro, Kalberer Anja, Stuetz Laura, Huber Sarina, Kaesermann Dominic, Guler Sabina and Wilhelm Matthias.” The correct spelling is “Prisca Eser, Pietro Calamai, Anja Kalberer, Laura Stuetz, Sarina Huber, Dominic Kaesermann, Sabina Guler and Matthias Wilhelm”.

The authors apologize for this error and state that this does not change the scientific conclusions of the article in any way. The original article has been updated.

