# Supplementary material for: Improved exercise ventilatory efficiency with nasal compared to oral breathing in cardiac patients
Source: Front Physiol. 2024 Aug 6;15:1380562. doi: 10.3389/fphys.2024.1380562 (PMC11334221; doi:10.3389/fphys.2024.1380562)
Supplement: Supplementary file 1 [file Table1.docx]

Supplement Table 1: Ventilatory and circulatory parameters during cardiopulmonary ramp test at rest, at VT1 and at peak exercise for each group separately. Shown are medians and first and third quartiles in round brackets.

|  | **CHF patients**  **(n = 15)** | **CCS patients**  **(n = 15)** | **Old, control subjects**  **(n = 12)** | **Young control subjects**  **(n = 15)** |
| --- | --- | --- | --- | --- |
| ***Resting*** |  |  |  |  |
| Ventilation [l∙min^-1^] | 15.3 (10.7, 16.1) | 14.5 (12.0, 16.8) | 14.0 (12.9, 15.4) | 13.4 (12.0, 16.7) |
| Ventilation [l∙kg^-1^ min^-1^] | 0.18 (0.15, 0.19) | 0.18 (0.16, 0.23) | 0.19 (0.17, 0.20) | 0.21 (0.20, 0.23) |
| Respiratory frequency [min^-1^] | 18.2 (14.1, 20.5) | 15.4 (13.3, 19.9) | 15.8 (14.5, 18.5) | 12.7 (12.0, 17.6) |
| Tidal volume [l] | 0.78 (0.72, 1.06) | 0.85 (0.80, 0.98) | 0.82 (0.69, 1.02) | 1.04 (0.84, 1.15) |
| Tidal volume [ml∙kg^-1^] | 10.8 (8.83, 12.3) | 11.5 (10.7, 12.6) | 12.2 (10.9, 13.1) | 15.4 (12.5, 17.5) |
| P_ET_CO_2_ [mmHg] | 26.0 (24.0, 27.0) | 25.0 (22.5, 26.0)* | 28.0 (27.5, 30.0) | 32.0 (30.0, 33.0) |
| Heart rate [bpm] | 65.0 (53.0, 73.5) | 62.0 (59.5, 79.0) | 65.0 (61.5, 73.0) | 76.0 (71.5, 94.0) |
| FVC [l] | 3.81 (3.14, 4.44) | 4.04 (3.57, 4.80) | 4.43 (3.58, 5.44) | 5.42 (4.51, 5.66) |
| FEV_1_ [l*min^-1^] | 2.82 (2.47, 3.51) | 2.96 (2.92, 3.43) | 3.00 (2.71, 4.06) | 4.20 (3.76, 4.57)* |
|  |  |  |  |  |
| ***At VT1*** |  |  |  |  |
| Power [watt] | 54.0 (45.5, 67.0) ^‡^ | 62.0 (56.0, 82.0)^†^ | 128 (103, 147) | 150 (126, 191) |
| V̇O_2_ [ml∙kg^-1^∙min^-1^] | 13.9 (11.2, 15.8) ^‡^ | 13.8 (12.0, 15.6) ^‡^ | 24.1 (21.3, 31.7) | 31.7 (23.5, 34.8) |
| Ventilation [l∙min^-1^] | 38.3 (32.4, 45.2) | 38.4 (33.1, 46.7) | 46.3 (40.5, 58.8) | 47.7 (41.4, 61.6) |
| Ventilation [l∙kg^-1^ min^-1^] | 0.49 (0.36, 0.56) ^†^ | 0.50 (0.44, 0.58)* | 0.67 (0.55, 0.78) | 0.72 (0.62, 0.94) |
| Respiratory frequency [min^-1^] | 23.9 (22.7, 25.4) | 22.0 (19.6, 25.1) | 23.0 (19.6, 23.5) | 24.1 (21.3, 26.3) |
| Tidal volume [l] | 1.50 (1.21, 1.96)* | 1.79 (1.54, 2.07) | 2.16 (1.96, 2.48) | 2.25 (1.89, 2.41) |
| Tidal volume [ml∙kg^-1^] | 18.8 (15.2, 22.9)* | 22.9 (18.6, 26.1)* | 32.0 (24.4, 39.6) | 30.9 (28.1, 35.7) |
| P_ET_CO_2_ [mmHg] | 31.0 (29.5, 33.0) ^‡^ | 31.0 (29.5, 32.0) ^‡^ | 39.0 (37.5, 42.0) | 41.0 (40.0, 44.0) |
| Heart rate [bpm] | 83.0 (77.0, 91.5) ^†^ | 92.0 (84.5, 99.5)* | 125 (109, 129) | 147 (137,158) |
|  |  |  |  |  |
| ***During ramp exercise*** |  |  |  |  |
| V̇_E_/V̇CO_2_-slope | 39.4 (37.0, 41.5) ^‡^ | 39.0 (38.3, 44.0) ^‡^ | 30.5 (26.4, 32.0) | 28.0 (25.8, 30.3) |
| Nadir V̇_E_/V̇CO_2_ | 36.5 (34.4, 39.2) ^‡^ | 38.0 (35.6, 39.1) ^‡^ | 28.1 (25.7, 29.0) | 26.3 (24.7, 27.3) |
|  |  |  |  |  |
| ***At peak exercise*** |  |  |  |  |
| Power [watt] | 110 (96.5, 125)^‡^ | 140 (121, 171)* | 211 (196, 235) | 299 (258, 343) |
| Power [watt∙kg^-1^] | 1.55 (1.27, 1.70)^‡^ | 1.78 (1.58, 1.90)* | 3.14 (2.69, 3.98) | 4.59 (3.89, 4.96) |
| V̇O_2_ [ml∙kg^-1^∙min^-1^] | 18.1 (15.1, 21.0)^‡^ | 20.9 (19.3, 23.0)^†^ | 36.9 (31.1, 41.7) | 46.8 (43.4, 52.3) |
| V̇O_2, max_ %predicted^a^ | 80.0 (71.5, 90.5)^‡^ | 87.0 (78.0, 102)^‡^ | 144 (130, 166) | 118 (114, 130) |
| Ventilation [l∙min^-1^] | 70.5 (63.0, 78.4)* | 84.1 (75.7, 107) | 95.3 (84.5, 107) | 131 (112, 162)* |
| Ventilation [l∙kg^-1^ min^-1^] | 0.93 (0.79, 1.08) ^†^ | 1.07 (1.00, 1.23) | 1.31 (1.21, 1.62) | 1.99 (1.82, 2.17)* |
| Respiratory frequency [min^-1^] | 37.6 (32.2, 40.8) | 38.3 (32.2, 49.8) | 38.4 (37.2, 42.5) | 54.9 (47.5, 60.6)^†^ |
| Tidal volume [l] | 1.88 (1.60, 2.38) | 2.45 (2.16, 2.50) | 2.36 (2.00, 2.90) | 2.19 (2.07, 2.86) |
| Tidal volume [ml∙kg^-1^] | 23.5 (20.4, 30.2)* | 30.6 (24.6, 33.1) | 33.9 (30.4, 38.2) | 35.5 (32.1, 39.2) |
| P_ET_CO_2_ [mmHg] | 27.0 (25.5, 29.0) ^‡^ | 27.0 (23.5, 28.0) ^‡^ | 35.0 (33.0, 37.5) | 33.0 (29.5, 35.5) |
| Heart rate [bpm] | 111 (92.0, 133) ^†^ | 126 (113, 142)* | 157 (151, 167) | 186 (183, 195)* |
| RER | 1.15 (1.10, 1.17) | 1.16 (1.08, 1.23) | 1.16 (1.10, 1.23) | 1.18 (1.16, 1.25) |

The following indices mark Benjamin-Hochberg adjusted p-value of post hoc Kruskal Wallis tests against old control subjects: * p<0.05; ^†^ p<0.01; ^‡^ p<0.001.

^a^, based on Wasserman, K., Hansen, J. E., Sue, D. Y., Whipp, B. J., & Casaburi, R. (1999). Principles of Exercise Testing and Interpretation: Including Pathophysiology and Clinical Applications. Lippincott Williams & Wilkins.

CHF, chronic heart failure; CCS, acute/chronic coronary syndrome; VT1, first ventilatory threshold; FVC, forced vital capacity; FEV_1_; forced expiratory volume in 1 s; V̇O_2_, oxygen uptake; P_ET_CO_2_, end-tidal carbon-dioxide partial pressure; RER, respiratory exchange ratio.

Supplement Table 2: Results from linear mixed models for parameters of breathing pattern and pulmonary gas exchange with effects for breathing modes and groups (incl. interaction), adjusted for testing order, sex, height, and weight.

|  |  | Estimate | standardised beta | 95% CI | p-value |
| --- | --- | --- | --- | --- | --- |
| ***V̇_E_/V̇CO_2_-ratio*** | Intercept | 32.71 | -0.23 | -0.75 – 0.28 | **<0.001** |
|  | CHFpatients | 8.12 | 1.28 | 0.77 – 1.79 | **<0.001** |
|  | CCSpatients | 7.94 | 1.25 | 0.74 – 1.76 | **<0.001** |
|  | Young,healthy | -2.28 | -0.36 | -0.85 – 0.14 | 0.154 |
|  | Mode:Nasal | -4.06 | -0.64 | -0.92 – -0.36 | **<0.001** |
|  | Order:2 | -0.61 | -0.10 | -0.22 – 0.03 | 0.127 |
|  | Male sex | -0.27 | -0.04 | -0.58 – 0.50 | 0.876 |
|  | Height | -0.23 | -0.04 | -0.28 – 0.21 | 0.771 |
|  | Weight | 0.10 | 0.02 | -0.20 – 0.23 | 0.882 |
|  | CHF patients x mode nasal | 0.57 | 0.09 | -0.27 – 0.45 | 0.623 |
|  | CCS patients x mode nasal | 0.86 | 0.13 | -0.23 – 0.50 | 0.463 |
|  | Young, healthy x mode nasal | 0.79 | 0.12 | -0.24 – 0.49 | 0.501 |
|  |  |  |  |  |  |
| ***Ventilation [l/min]*** | Intercept | 52.13 | 0.03 | -0.55 – 0.61 | **<0.001** |
|  | CHF patients | -10.36 | -0.90 | -1.48 – -0.32 | **0.003** |
|  | CCS patients | -4.11 | -0.36 | -0.93 – 0.22 | 0.222 |
|  | Young, healthy | 8.08 | 0.70 | 0.14 – 1.26 | **0.015** |
|  | Mode: Nasal | -5.95 | -0.52 | -0.86 – -0.17 | **0.004** |
|  | Order: 2 | -0.42 | -0.04 | -0.19 – 0.12 | 0.643 |
|  | Male sex | 5.21 | 0.45 | -0.15 – 1.05 | 0.139 |
|  | Height | 3.45 | 0.30 | 0.03 – 0.57 | **0.033** |
|  | Weight | 0.56 | 0.05 | -0.19 – 0.29 | 0.690 |
|  | CHF patients x mode nasal | 4.56 | 0.40 | -0.06 – 0.85 | 0.088 |
|  | CCS patients x mode nasal | 1.73 | 0.15 | -0.30 – 0.60 | 0.513 |
|  | Young, healthy x mode nasal | 0.63 | 0.05 | -0.40 – 0.51 | 0.812 |
|  |  |  |  |  |  |
| ***Breathing rate [bpm]*** | Intercept | 25.55 | 0.02 | -0.68 – 0.72 | **<0.001** |
|  | CHF patients | 3.18 | 0.58 | -0.13 – 1.29 | 0.107 |
|  | CCS patients | 2.16 | 0.40 | -0.31 – 1.10 | 0.268 |
|  | Young, healthy | 4.53 | 0.83 | 0.14 – 1.52 | **0.019** |
|  | Mode: Nasal | -4.26 | -0.78 | -1.28 – -0.28 | **0.003** |
|  | Order: 2 | -0.14 | -0.03 | -0.25 – 0.20 | 0.822 |
|  | Male sex | -0.50 | -0.09 | -0.80 – 0.62 | 0.798 |
|  | Height | -1.32 | -0.24 | -0.57 – 0.08 | 0.141 |
|  | Weight | -0.19 | -0.04 | -0.32 – 0.25 | 0.804 |
|  | CHF patients x mode nasal | -0.69 | -0.13 | -0.79 – 0.53 | 0.705 |
|  | CCS patients x mode nasal | 0.62 | 0.11 | -0.55 – 0.77 | 0.735 |
|  | Young, healthy x mode nasal | -1.63 | -0.30 | -0.96 – 0.37 | 0.376 |
|  |  |  |  |  |  |
| ***Tidal volume [l]*** | Intercept | 2.12 | -0.02 | -0.65 – 0.61 | **<0.001** |
|  | CHF patients | -0.62 | -0.96 | -1.59 – -0.33 | **0.003** |
|  | CCS patients | -0.38 | -0.59 | -1.21 – 0.03 | 0.062 |
|  | Young, healthy | -0.08 | -0.13 | -0.74 – 0.48 | 0.670 |
|  | Mode: Nasal | 0.19 | 0.29 | -0.04 – 0.63 | 0.083 |
|  | Order: 2 | 0.00 | 0.00 | -0.15 – 0.14 | 0.960 |
|  | Male sex | 0.25 | 0.39 | -0.27 – 1.05 | 0.243 |
|  | Height | 0.22 | 0.35 | 0.04 – 0.65 | **0.025** |
|  | Weight | 0.05 | 0.07 | -0.19 – 0.33 | 0.599 |
|  | CHF patients x mode nasal | 0.04 | 0.07 | -0.37 – 0.51 | 0.752 |
|  | CCS patients x mode nasal | -0.04 | -0.06 | -0.50 – 0.38 | 0.791 |
|  | Young, healthy x mode nasal | 0.24 | 0.37 | -0.07 – 0.81 | 0.102 |
|  |  |  |  |  |  |
| ***P_ET_CO_2_ [mmHg]*** | Intercept | 35.62 | 0.13 | -0.43 – 0.68 | **<0.001** |
|  | CHF patients | -6.59 | -1.11 | -1.66 – -0.56 | **<0.001** |
|  | CCS patients | -6.55 | -1.10 | -1.65 – -0.56 | **<0.001** |
|  | Young, healthy | 1.46 | 0.25 | -0.29 – 0.78 | 0.362 |
|  | Mode: Nasal | 5.26 | 0.88 | 0.58 – 1.19 | **<0.001** |
|  | Order: 2 | 0.79 | 0.13 | -0.00 – 0.27 | 0.054 |
|  | Male sex | 0.18 | 0.03 | -0.55 – 0.61 | 0.919 |
|  | Height | 0.33 | 0.06 | -0.21 – 0.32 | 0.676 |
|  | Weight | -0.03 | -0.01 | -0.24 – 0.22 | 0.965 |
|  | CHF patients x mode nasal | -2.48 | -0.42 | -0.81 – -0.02 | **0.040** |
|  | CCS patients x mode nasal | -2.19 | -0.37 | -0.77 – 0.03 | 0.070 |
|  | Young, healthy x mode nasal | -1.09 | -0.18 | -0.58 – 0.22 | 0.367 |
|  |  |  |  |  |  |
| ***P_ET_O_2_ [mmHg]*** | Intercept | 100.05 | -0.34 | -0.92 – 0.24 | **<0.001** |
|  | CHF patients | 9.36 | 1.36 | 0.78 – 1.93 | **<0.001** |
|  | CCS patients | 8.88 | 1.29 | 0.71 – 1.86 | **<0.001** |
|  | Young, healthy | 1.50 | 0.22 | -0.34 – 0.78 | 0.445 |
|  | Mode: Nasal | -5.56 | -0.80 | -1.14 – -0.47 | **<0.001** |
|  | Order: 2 | -1.21 | -0.17 | -0.33 – -0.02 | **0.024** |
|  | Male sex | -0.39 | -0.06 | -0.66 – 0.55 | 0.854 |
|  | Height | -0.77 | -0.11 | -0.39 – 0.16 | 0.427 |
|  | Weight | -0.42 | -0.06 | -0.30 – 0.18 | 0.617 |
|  | CHF patients x mode nasal | 2.23 | 0.32 | -0.12 – 0.77 | 0.154 |
|  | CCS patients x mode nasal | 2.23 | 0.32 | -0.12 – 0.77 | 0.154 |
|  | Young, healthy x mode nasal | 0.77 | 0.11 | -0.34 – 0.56 | 0.625 |
|  |  |  |  |  |  |
| ***RSBI*** | Intercept | 13.75 | 0.03 | -0.56 – 0.62 | **<0.001** |
|  | CHF patients | 7.45 | 1.17 | 0.57 – 1.78 | **<0.001** |
|  | CCS patients | 3.28 | 0.52 | -0.09 – 1.12 | 0.092 |
|  | Young, healthy | 2.45 | 0.39 | -0.21 – 0.98 | 0.198 |
|  | Mode: Nasal | -2.59 | -0.41 | -0.87 – 0.06 | 0.084 |
|  | Order: 2 | 0.03 | 0.01 | -0.20 – 0.21 | 0.960 |
|  | Male sex | -2.59 | -0.41 | -1.00 – 0.18 | 0.174 |
|  | Height | -2.36 | -0.37 | -0.64 – -0.10 | **0.008** |
|  | Weight | -0.43 | -0.07 | -0.30 – 0.17 | 0.573 |
|  | CHF patients x mode nasal | -3.59 | -0.57 | -1.18 – 0.04 | 0.069 |
|  | CCS patients x mode nasal | 0.05 | 0.01 | -0.60 – 0.62 | 0.978 |
|  | Young, healthy x mode nasal | -1.22 | -0.19 | -0.81 – 0.42 | 0.537 |
|  |  |  |  |  |  |
| ***V̇O_2_ [ml/kg/min]*** | Intercept | 25.91 | 0.31 | -0.10 – 0.71 | **<0.001** |
|  | CHF patients | -10.07 | -1.10 | -1.50 – -0.71 | **<0.001** |
|  | CCS patients | -7.99 | -0.88 | -1.27 – -0.48 | **<0.001** |
|  | Young, healthy | 5.54 | 0.61 | 0.22 – 0.99 | **0.002** |
|  | Mode: Nasal | 0.69 | 0.08 | -0.08 – 0.23 | 0.320 |
|  | Order: 2 | 0.67 | 0.07 | 0.01 – 0.14 | **0.034** |
|  | Male sex | -0.35 | -0.04 | -0.47 – 0.39 | 0.862 |
|  | Height | 1.85 | 0.20 | 0.01 – 0.40 | **0.044** |
|  | Weight | -2.72 | -0.30 | -0.47 – -0.13 | **0.001** |
|  | CHF patients x mode nasal | 0.33 | 0.04 | -0.16 – 0.23 | 0.722 |
|  | CCS patients x mode nasal | -0.47 | -0.05 | -0.25 – 0.15 | 0.609 |
|  | Young, healthy x mode nasal | 1.01 | 0.11 | -0.09 – 0.31 | 0.275 |
|  |  |  |  |  |  |
|  |  |  |  |  |  |
| **HR [bpm]** | Intercept | 124.98 | 0.22 | -0.23 – 0.67 | **<0.001** |
|  | CHF patients | -36.84 | -1.35 | -1.79 – -0.92 | **<0.001** |
|  | CCS patients | -27.12 | -1.00 | -1.43 – -0.56 | **<0.001** |
|  | Young, healthy | 21.57 | 0.79 | 0.37 – 1.21 | **<0.001** |
|  | Mode: Nasal | 0.86 | 0.03 | -0.09 – 0.16 | 0.620 |
|  | Order: 2 | 1.89 | 0.07 | 0.01 – 0.13 | **0.016** |
|  | Male sex | 4.73 | 0.17 | -0.31 – 0.66 | 0.475 |
|  | Height | -2.06 | -0.08 | -0.30 – 0.14 | 0.498 |
|  | Weight | -2.71 | -0.10 | -0.29 – 0.09 | 0.307 |
|  | CHF patients x mode nasal | 2.99 | 0.11 | -0.06 – 0.28 | 0.194 |
|  | CCS patients x mode nasal | 2.79 | 0.10 | -0.06 – 0.27 | 0.224 |
|  | Young, healthy x mode nasal | -0.49 | -0.02 | -0.19 – 0.15 | 0.832 |
|  |  |  |  |  |  |
| ***V̇O_2_*/HR [ml/beat]** | Intercept | 14.49 | 0.14 | -0.50 – 0.77 | **<0.001** |
|  | CHF patients | -1.84 | -0.54 | -1.16 – 0.08 | 0.088 |
|  | CCS patients | -1.72 | -0.50 | -1.12 – 0.11 | 0.108 |
|  | Young, healthy | 0.23 | 0.07 | -0.53 – 0.67 | 0.821 |
|  | Mode: Nasal | 0.35 | 0.10 | -0.12 – 0.33 | 0.369 |
|  | Order: 2 | 0.14 | 0.04 | -0.06 – 0.14 | 0.433 |
|  | Male sex | 0.30 | 0.09 | -0.59 – 0.76 | 0.800 |
|  | Height | 1.61 | 0.47 | 0.16 – 0.78 | **0.003** |
|  | Weight | 0.79 | 0.23 | -0.04 – 0.50 | 0.094 |
|  | CHF patients x mode nasal | -0.03 | -0.01 | -0.30 – 0.29 | 0.957 |
|  | CCS patients x mode nasal | -0.53 | -0.15 | -0.45 – 0.14 | 0.303 |
|  | Young, healthy x mode nasal | 0.35 | 0.10 | -0.19 – 0.40 | 0.491 |

* 95% confidence interval of standardised beta

CCS, chronic coronary syndrome; VT1, first ventilatory threshold; V̇e, minute ventilation; f_R,_ breathing frequency; V_T_, tidal volume*;* P_ET_CO_2,_ end-tidal partial pressure of CO_2_; P_ET_O_2_, end-tidal partial pressure of O_2_; V̇O_2_, oxygen consumption; HR, heart rate
