# Supplementary figures and images for: Improved exercise ventilatory efficiency with nasal compared to oral breathing in cardiac patients
Source: Front Physiol. 2024 Aug 6;15:1380562. doi: 10.3389/fphys.2024.1380562 (PMC11334221; doi:10.3389/fphys.2024.1380562)

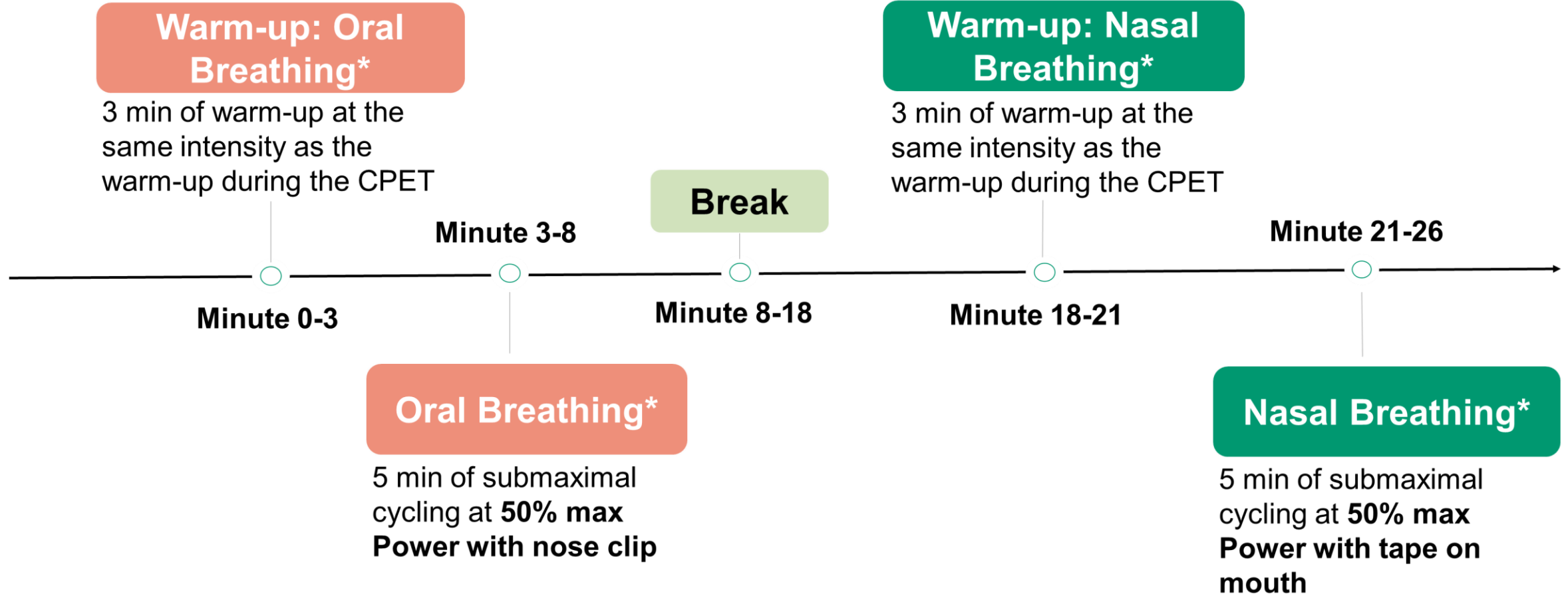

Supplement: Supplementary file 2 [file Image1.pdf]
